# Properties and Structure of Thermoplastic Polyvinyl Alcohol/Polyamide Sea-Island Fibers

**DOI:** 10.3390/polym15092071

**Published:** 2023-04-27

**Authors:** Bing Song, Yang Cao, Liang Wang, Yake Shen, Xiaoming Qian

**Affiliations:** 1 School of Textiles Science and Engineering, Tiangong University, Tianjin 300387, China; 2 Mingxin Xuteng (Jiangsu) Innovation Research Institute Limited Company, Xinyi 221433, China

**Keywords:** sea-island fiber, conjugated melt spinning, water-splitting, ultra-fine fiber, thermoplastic polyvinyl alcohol

## Abstract

Ultra-fine fibers derived from sea-island fibers have attracted great attention due to their excellent overall performance. However, green and efficient splitting of sea-island fibers is still a challenging task. In this work, thermoplastic polyvinyl alcohol (TPVA) was prepared by the physical blending of plasticizer. The modified TPVA showed a high decomposition temperature (285 °C) and a wide thermoplastic processing window. This made TPVA match well with polyamide 6 (PA6) to form conjugated melts at 250 °C. Corresponding PVA/PA6 sea-island fibers were first reported to realize water-splitting instead of alkali-extraction of “sea” polymers. The effects of sea/island mass ratios and different spinning speeds on the properties of PVA/PA6 sea-island pre-oriented yarn (POY) were investigated. A higher spinning speed enhanced the orientation-induced crystalline behavior of fiber, therefore increasing the tensile strength of fibers. As the increase of spinning speed from 1000 to 1500 m/min, the crystalline degree of corresponding POYs increased from 9.9 to 14.3%. The plasticizer in PVA did not diffuse to the PA matrix during spinning. However, PVA could induce the crystallization of PA6 via interfacial hydrogen bonding. When the spinning speed was 1500 m/min, and PVA/PA6 was 7:3, the tensile strength reached the highest value of 1.67 cN/dtex. The uniform diameters of ultra-fine PA6 fibers (2–5 μm) were obtained by an environment-friendly water-splitting process. The “sea” phase (TPVA) in sea-island fiber could be removed quickly by boiling water treatment in 3 min. This green and energy-saving sea-island fiber splitting technique is of great significance in reducing CO_2_ emissions during the preparation of super-fine fibers.

## 1. Introduction

Ultra-fine fibers exist widely in nature, such as spider silk, chamois leather, and peach skin [1]. They have recently received great attention due to their special properties, including high tenacity and touching comfort. Ultra-fine fiber is generally defined to have a diameter of less than 5 µm or 0.55 dtex. By virtue of small fineness, the rigidity of fiber decreases exponentially [2]. Corresponding products, such as synthetic leather, show a higher added value owing to novel tactile, visual aesthetics, and drapeability, as well as softness [3,4,5]. Ultra-fine fibers also exhibit a large specific surface area, so the filters made by ultra-fine fibers normally show excellent absorption properties. Moreover, the packing density of filters increases with the decrease in fiber diameter, increasing the filtration properties of materials.

Ultra-fine fiber could be directly obtained via electrospinning or a two-step spinning method such as sea-island spinning. In industry, they are usually manufactured by a commercially applicable process. Sea-island melt-spinning is currently the most developed technique in terms of available production scale. Two immiscible or partially miscible polymer melting blends are extruded through a spinneret. The melt flow comprises a dispersed phase and a matrix phase, which transforms into “islands” embedded in a “sea”. Once the “sea” ingredients are removed during post-splitting, continuous filaments or random staple yarns are obtained [6]. The most commonly seen commercial products made of ultra-fine fibers are synthetic leathers composed by polyurethane coating of ultra-fine fiber non-woven mats.

Sea-island spinning is divided into blending, spinning, and conjugated spinning. Blending sea-island spinning usually generates staple yarn of ultra-fine fibers. The uneven dimension of fiber in random staple yarn causes an inadequate tactile and visual aesthetics of synthetic leather. In contrast, in conjugated sea-island spinning, the dispersed phase is designed as an individual “island” in the matrix (“sea”) to make ultra-fine filament with a uniform diameter. It was verified that the textiles comprised of uniform ultra-fine fibers had superior physical properties, such as high mechanical strength and color fastness [7,8,9]. The dimension of desired ultra-fine fibers could be precisely controlled with the aid of melt or plastic flow [10,11].

“Sea” polymers at least match well the melt-spinning temperature (>260 °C) and corresponding rheological behavior of “island” polymer (i.e., polyester or polyamide) [12,13]. Copolyester (COPET), which possesses an easily soluble characteristic in dilute alkali solution, is usually chosen as the “sea” polymer in conjugated sea-island spinning [14,15]. However, the use of alkalis brings a large quantity of wastewater. Under the policy of clean production, it is desired to develop sea-island fiber that is susceptible to water weight reduction. On the other side, COPET usually requires an alkali extraction for 30 min. A high-productivity manufacturing process asks for the rapid removal of the sea component. However, regarding this issue, to our best knowledge, only rare reports have appeared in research or industrial practice.

Polyvinyl alcohol (PVA) is a type of water-soluble and biodegradable polymer that is commonly used in making fibers and films [16,17,18,19]. Its thermoplastic modification could be realized via chemical copolymerization or physical blending [20]. The latter strategy is more frequently reported owing to the facile operation and clean production [21,22,23]. Generally, various plasticizers are blended with commercial PVA pellets in a two-screw extruder. In this process, the macromolecular tacticity of PVA is broken by reducing inter-molecular interaction [24,25,26,27,28,29]. The rheological behavior and spinnability of modified PVA were frequently discussed [30,31]. However, these works have not improved the thermal stability of thermoplastic PVA that normally decomposes before 250 °C. The reported thermoplastic PVA (TPVA) can not fulfill the sea-island spinning to prepare high-performance ultra-fine fibers.

In this work, TPVA with a high decomposition temperature (>270 °C) is first prepared and then used as a sea component for sea-island melt-spinning. They were later removed from sea-island fiber through boiling water treatment. Ultra-fine PA6 fibers were then obtained by an efficient and environmentally friendly water-splitting process. Compared to the industrial COPET/PA6 sea-island fibers that were split by hot alkali solution (95 °C) for 30 min, as-prepared PVA/PA6 sea-island fibers were totally split in boiling water in 3 min. The effect of the ratio of island/sea components and spinning speed on the morphologies, crystallization behavior, and tensile properties of pre-oriented yarn (POY) were investigated, respectively. The involved energy-saving, efficient and environment-friendly technique paves the way to prepare high-performance ultra-fine fibers.

## 2. Materials and Methods 

### 2.1. Materials

Polyvinyl vinyl alcohol (PVA) pellets were supplied by the Anhui Vinylon factory of Sinopec Co. Ltd., Bengbu, China. It possessed a degree of saponification of 80 mol% and a degree of polymerization of 900 (trademark 080-09). Sorbitol ether was purchased from Aladdin agent (Shanghai, China) Co. PA6 pellets were purchased from Yizhen Sinopec Co., Ltd. Antioxidant (MIANOX445) was obtained from Xince (Changzhou, China) Polymer Co., Ltd.

### 2.2. Thermoplastic Modification of PVA

To enhance the melting processability, the raw PVA was plasticized using sorbitol ether (8 wt%) and 2 wt% of antioxidant. All components were mixed to homogeneously distribute the plasticizer in the polymer and then matured for 2 h at 80 °C. A two-screw (RXT2, Ruiya Nanjing, China) with an L/D ratio of 40 and a speed of 160 rpm was employed. The processing temperature was controlled at different zones of the extruder to obtain an ascending temperature profile from 110 °C to 180 °C. That modified polymer was named thermoplastic PVA (TPVA).

### 2.3. Spinning of Sea-Island Pre-Oriented Yarn

The granulated TPVA and PA6 were dried at 90 °C in a vacuum oven for 24 h before melt-spinning. The sea-island spinning was conducted on a conjugated bicomponent spinning machine (Fangchen, Zhibo, China). TPVA was used as a “sea” ingredient and extruded by extruder A with an ascending temperature at different zones (210 °C, 220 °C, 225 °C, 230 °C, 235 °C, and 240 °C). PA6 (“island” polymer) was sequenced out by extruder B (230 °C, 235 °C, 240 °C, 245 °C, and 250 °C). The temperature of the die was set to 250°C. The sea/island component mass ratio was set as 5:5, 4:6, 3:7, and 2:8, respectively. The rolling speed was set as 1000, 1200, 1500, and 2000 m/min, respectively.

### 2.4. Characterization

Transmission-FTIR spectra of PVA and TPVA were recorded using a spectrometer (Nicolet iS50, Thermo Fisher Scientific, Shanghai, China) in the spectral range from 400 to 4000 cm^−1^ based on 30 scans. X-ray diffraction (XRD) measurements were carried out on a D8 Discover X-ray diffractometer (Bruker, Billerica, MA, USA) using Cu–Ka radiation. Thermogravimetric analysis (TGA) was carried out using a STA449F3 thermal analyzer (Netzsch, Bavaria, Germany) at a heating rate of 10 °C/min from 30 °C to 800 °C under an N_2_ atmosphere. Differential scanning calorimetry (DSC) curves were recorded on 200F3 instruments (Netzsch). Ten milligrams of specimens were first heated from 30 to 250 °C at a heating rising ramp of 10 °C/min and held at 250 °C for 1 min. The samples were then cooled to 30 °C at the same temperature ramp. The cross-sectional morphology of the prepared sea-island fiber was examined by a Hitachi-S4800N scanning electron microscope. Before observation, sea-island fibers were first cut using a Haas section and then sputtered with a thin layer of gold. Tensile properties of as-prepared POY were studied using a Universal Testing Machine (Instron 3369, Norwood, MA, USA) according to GB/T 14337-2008. The crosshead speed and distance were set at 40 mm/min and 20 mm, respectively.

## 3. Results and Discussion

### 3.1. Chemical Structure and Thermal Behavior of TPVA

**Figure 1:**
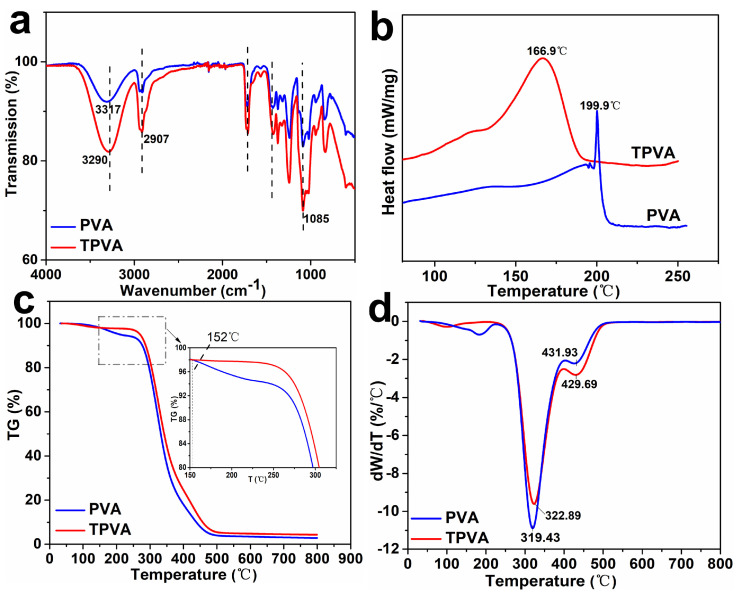
PVA FTIR (**a**), DSC (**b**), weight loss (**c**) and DTG (**d**) curves before and after plasticization.

The chemical structure and thermal behavior of modified PVA (TPVA) were analyzed, as shown in Figure 1. The peak at 1085 cm^−1^ and 2907 cm^−1^ contributes to C–O stretching and C–H stretching in FTIR spectra, respectively. Raw PVA showed an absorption peak at 3316 cm^−1^ that ascribed to O–H stretching, as shown in Figure 1a. This broad peak shifted to 3288 cm^−1^ with the addition of a plasticizer, which decreased intra-molecular hydrogen bonding in PVA. This band also increased in intensity due to the increase in the quantity of the –OH group from polyol [32]. Correspondingly, a broader melting peak was observed in the DSC curve of TPVA (Figure 1b). The melting point of TPVA (T_m_) decreased to 166.9 °C by adding polyol. It was because the interaction between PVA molecules decreased, which weakened the crystalline ability of PVA.

It was investigated that the weight and derived thermal gravity (DTG) changed with temperature. Raw PVA began to decompose at 181 °C (T_d_) due to the evaporation of bonded water [24]. TPVA showed a much higher initial decomposition temperature. It remained thermo-stable even when the temperature was higher than 280 °C (Figure 1c). On the other side, even though the highest temperature at maximum decomposition rate (T_dmax_) only improved by 3.4 °C, the highest decomposition rate decreased from 11 to 9.5%/°C after plasticization, as shown in Figure 1d. New and stronger hydrogen bonding were formed between PVA and polyol. This resulted in the reduced chain mobility of the PVA chain and increased thermal stability. The larger difference between T_m_ and T_d_ indicated the thermoplastic processing property of PVA was significantly improved after plasticization.

### 3.2. Effect of Sea-Island Ratio and Spinning Speed on Microstructure

**Figure 2:**
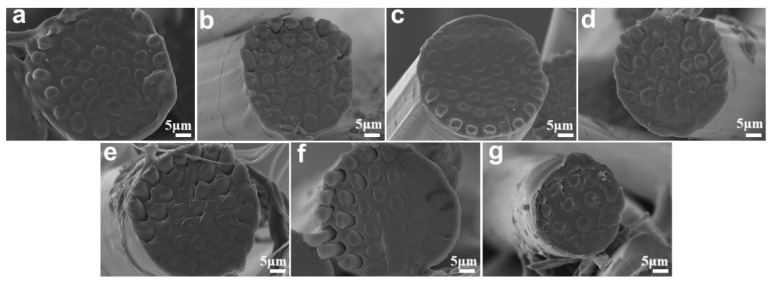
(**a**–**d**) PVA/PA6 2:8, PVA/PA6 3:7, PVA/PA6 4:6, and PVA/PA6 5:5 at 1200 m/min; (**e**–**g**) PVA/PA6 3:7 at 1000 m/min, 1500 m/min, and 2000 m/min.

Sea-island fibers were prepared from different sea/island mass ratios and spinning speeds, and the morphologies are shown in Figure 2. A clear interfacial phase was observed in all obtained fibers. It is possibly caused by the prominent difference in crystalline behavior between PVA and PA6 [30,33]. The sea/island mass ratio did not affect the dimension of the fiber. However, the “sea” part occupied more space with the increase of PA6′ ratio, as shown in Figure 2a–d. The diameter of round seaisland fiber decreased with the increase in spinning speed, as seen in Figure 2e–g. However, the skin of the sea/island began to break up when the speed reached 2000 m/min (Figure 2g). This is because PVA melts break under a high stretching speed.

**Figure 3:**
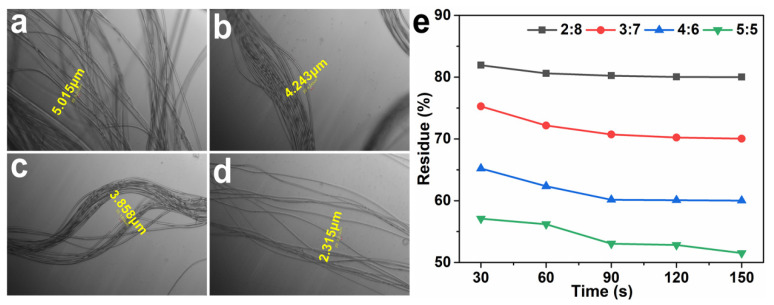
Optical photos of super-fine fiber obtained from different spinning speeds (**a**) 1000, (**b**) 1200, (**c**) 1500, and (**d**) 2000 m/min. (**e**) Weight residue vs. splitting time in boiling water.

The splitting behavior of sea-island fiber was studied, as shown in Figure 3. Sea-island fiber turned to super-fine fiber with a diameter of 2–5 μm after boiling water treatment, as seen in Figure 3a–d. The diameter of ultra-fine PA6 fibers decreased with the increase in spinning speed. Two minutes of water-splitting nearly removed sea polymer, as shown in Figure 3e. Moreover, with the increase of “sea” polymer in sea-island fibers, the splitting tine of sea-island fibers reduced. As a comparison, the industrial splitting of COPET/PA6 sea-island fiber usually costs 30 min under 5 wt% NaOH solution at 95 °C [12]. It suggests a cleaner splitting process that could realize the reduction of energy usage and wastewater.

### 3.3. XRD Analysis of Sea-Island Fiber

**Figure 4:**
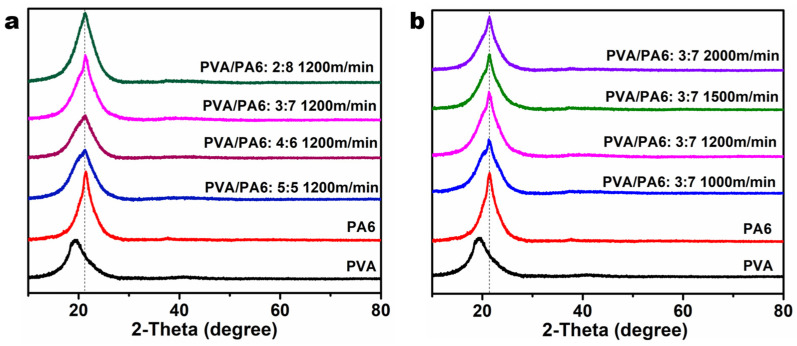
XRD patterns of sea-island fibers prepared from different spinning speeds (**a**) and sea/island mass ratios (**b**).

The XRD patterns (Figure 4) were measured to understand the microstructures of as-prepared sea-island fibers. The interplanar spacing (L_c_) and the crystalline size (D) were calculated from the Bragg angle and half bandwidth, as shown in Table 1. PVA and PA6 fiber showed a peak at 19.4° and 21.4°, respectively. They were ascribed to (101) and (200) crystal planes, respectively [34,35]. Regarding the sea-island fibers, the diffractive peak turned from broad to sharp with the increase of island polymer content (Figure 4a). Especially when the sea/island mass ratio reached 2:8, it disappeared the diffractive peak of the (101) plane. This indicated that the crystalline behavior of TPVA was affected by PA6 melting flow. To be mentioned, the L_c_ of (200) in pure PA6 fiber was greater than the one in each sea-island fiber. This is possibly the case because the PVA or polyol diffusion affected the packing of PA6 molecules on the interface.

There was no significant change in the crystalline size of (200) from PA6 (Figure 4b) when increasing the spinning speed. However, it decreased the crystalline size of (101) from TPVA from 0.7 to 0.2 Å, as seen in Table 1. The possible reason is that the folded chain thickness decreased by higher stretching, especially when the spinning speed exceeds 2000 m/min.

### 3.4. Thermal Analysis of Sea-Island Fibers

**Figure 5:**
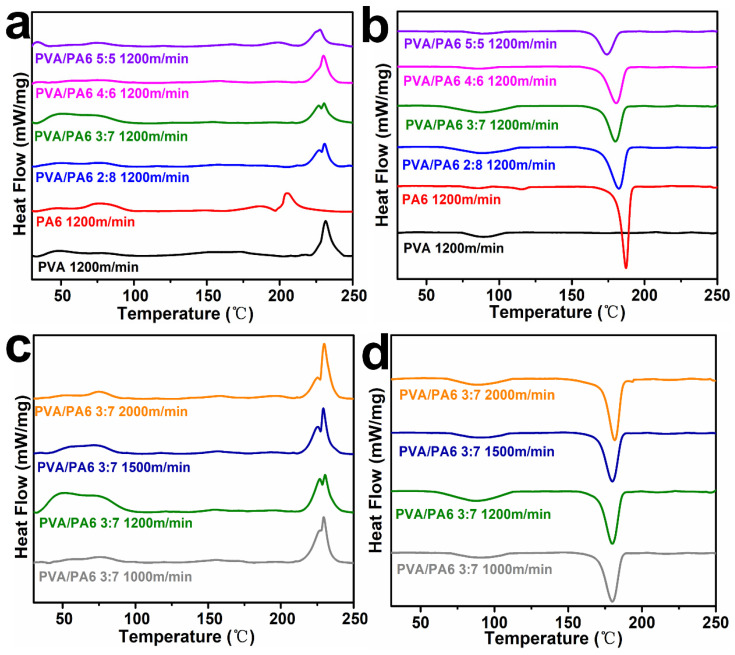
The first heating (**a**,**c**) and cooling (**b**,**d**) DSC patterns of sea-island fibers were prepared by various sea/island mass ratios and spinning speeds, respectively.

The thermal properties of as-prepared fibers were studied by DSC analysis. The corresponding first heating curve and the first cooling patterns are shown in Figure 5. Crystallinity degree (X%), crystallization temperature (T_c_), and melting temperature (T_m_) are included in Table 2. The crystalline degree (X%) was calculated according to X = ΔH/ΔH_f_, where ΔH and ∆H_f_ were the crystallization enthalpy of the sample and 100% crystalline PVA (138.6 J/g) [30], respectively. T_m_ of sea-island fiber increased with the increase in spinning speed (Figure 5b). This was because the higher speed generated a higher orientation of macromolecules, which helped the packing of the polymer chain and increased the thickness of lamellas [36]. The orientation induced crystallization: therefore, the crystalline degree of fiber increased from 9.9% to 14%. However, the prepared pre-oriented fibers sustained a low crystalline degree. This is because the prepared POY lacks thermal fixing. PVA and PA6 showed a T_m_ of 231.8 and 205.6 °C, respectively. Sea-island fibers exhibited merged melting peaks. When the sea/island mass ratio went beyond 4:6 (Figure 5a), a unique melting peak was observed. It indicated that PVA induced crystallization of PA6 during spinning. Therefore, the melting point of PA6 increased in sea-island fibers. PVA enriched with –OH group, inducing interfacial crystallization via hydrogen bonding during spinning.

In cooling curves (Figure 5b,d), PVA did not show any crystallization behavior due to the existence of a large quantity of plasticizer, which prevented the crystallization of PVA during cooling. The crystalline ability of blended melt reduced with the content of PVA. Nevertheless, the crystalline temperature did not show a significant change. It suggested the polyols did not diffuse into PA6 and only affected the crystallization of PA6 in the phase interface.

### 3.5. Mechanical Properties of Sea-Island Fibers

**Figure 6:**
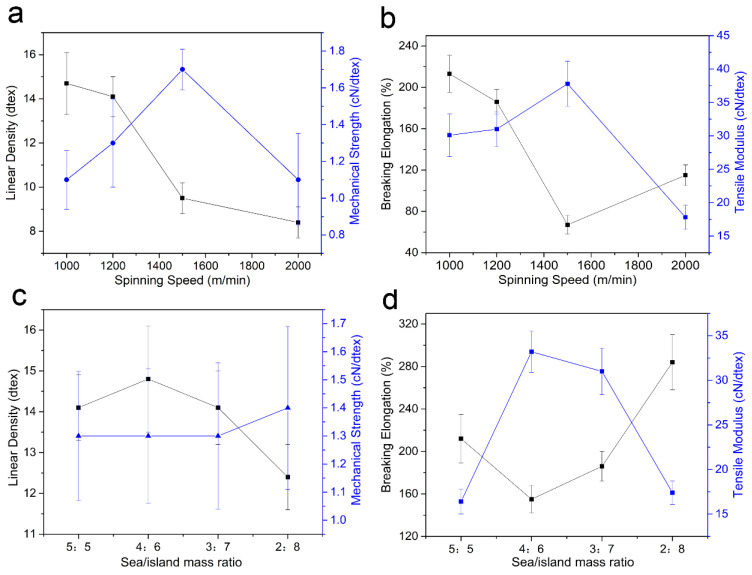
Tensile properties of as-prepared PVA–PA6 sea-island POY: (**a**) linear density and mechanical strength Vs spinning speed; (**b**) breaking elongation and tensile modulus Vs spinning speed; (**c**) linear density and mechanical strength Vs sea/island ratio; (**d**) breaking elongation and tensile modulus Vs sea/island ratio.

The tensile properties were studied, and the results are shown in Figure 6. Sea-island mass ratio did not affect the fiber’s dimension significantly (Figure 6c). Increasing the weight ratio of PA6 from 5:5 to 4:6, the breaking elongation of fibers initially decreased due to the increase of crystalline degree. It then increased due to the intrinsically excellent ductility of PA6 when PA6 occupied more than 60% [37], as seen in Figure 6d. When the mass ratio of the sea/island phase reached 2:8, the breaking elongation of POY displayed as high as 300%. Owing to the low crystallization degree of POY, as-prepared fiber exhibited low mechanical strength (<1.7 cN/dtex) and high breaking elongation (>60%). A higher spinning speed, and a higher crystalline degree, generate a higher mechanical strength and elastic moduli of sea-island fibers. When the spinning speed increased from 1000 to 1500 m/min, the mechanical strength of sea-island POYs increased from 1.05 to 1.7 cN/dtex. However, when the spinning speed exceeded 2000 m/min, cracked weakness in sea-island fibers decreased the mechanical properties of fibers to 1.1 cN/dtex, as shown in Figure 6a. Correspondingly, the tensile moduli of fibers followed the tendency of mechanical strength (Figure 6b). This was relative to the crystalline change in sea-island fibers.

## 4. Conclusions

PVA/PA6 sea/island fibers were first prepared in this work. The modified PVA had excellent thermal stability and showed an initial decomposition temperature of 285 °C. This ensured a conjugated spinning of PVA/PA6 blended melts. The effect of the mass ratio of the sea/island phase and spinning speed on the crystalline behavior and tensile properties of POY were discussed. Generally, as-prepared sea-island POY showed a low crystalline degree. Therefore, they lack mechanical strength. The maximum crystalline degree reached 14.3%, and the highest tensile strength was 1.67 cN/dtex. A higher spinning speed induced the orientation of macromolecules and improved the crystalline degree of fiber. The plasticizer in PVA did not diffuse to the PA6 matrix during spinning. PVA could induce the crystallization of PA6 through interfacial bonding. The as-prepared sea-island fibers turned into super-fine fibers with a diameter of 2–5 μm in 3 min by water-splitting. TPVA, in this study, as the sacrificial phase, could be a promising substitute in place of COPET to eliminate issues associated with an environment-unfriendly splitting process. It also provided a facile and economical approach to the high throughput fabrication of fine fibers.

## Figures and Tables

**Table 1:** Crystalline parameters of as-prepared sea-island POY.

Sample	101 (°)	200 (°)	D_101_ (Å)	D_200_ (Å)	L_c101_ (Å)	L_c200_ (Å)
PVA–1200 m/min	19.4	–	0.3	–	1.5	–
PA6–1200 m/min	–	21.4	–	0.5	–	1.4
PVA/PA6 2:8–1200 m/min	–	21.2	–	0.5	–	1.1
PVA/PA6 3:7–1200 m/min	20.4	21.3	0.7	0.5	0.8	1.2
PVA/PA6 4:6–1200 m/min	20	21.3	0.6	0.7	0.8	1.2
PVA/PA6 5:5–1200 m/min	19.5	21.3	0.2	0.6	1.3	1
PVA/PA6 3:7–1000 m/min	20.1	21.5	0.7	0.5	0.8	1.5
PVA/PA6 3:7–1500 m/min	20.5	21.3	0.4	0.5	0.8	1.1
PVA/PA6 3:7–2000 m/min	19.9	21.3	0.2	0.5	0.9	1.2

**Table 2:** Crystalline degree and thermal behavior of as-prepared sea-island POY.

Sample	T_m_ (°C) ^a^	ΔH (J/g)	X (%)	T_c_ (°C) ^b^
PVA–1200 m/min	295.6	17.6	10.4	–
PA6–1200 m/min	231.8	26.7	14.1	187.2
PVA/PA6 2:8–1200 m/min	230.8	17.8	9.6	182.3
PVA/PA6 3:7–1200 m/min	230.3	22.1	12.1	180.4
PVA/PA6 4:6–1200 m/min	230.1	26.3	14.3	180.6
PVA/PA6 5:5–1200 m/min	227.5	15.6	9.3	173.3
PVA/PA6 3:7–1000 m/min	229.4	18.1	9.9	179.7
PVA/PA6 3:7–1500 m/min	230.5	22.8	12.4	180.3
PVA/PA6 3:7–2000 m/min	230.6	25.6	14	181.7

^a^ T_m_ is obtained from the first heating curve. ^b^ T_c_ is taken from the first cooling pattern. “–” means there is no crystalline temperature.

## Data Availability

Data will be available on request.
